# Male esthetic genital surgery: recommendations and gaps to be filled

**DOI:** 10.1038/s41443-022-00556-6

**Published:** 2022-04-05

**Authors:** Carlo Bettocchi, Andrea Alberto Checchia, Ugo Giovanni Falagario, Anna Ricapito, Gian Maria Busetto, Luigi Cormio, Giuseppe Carrieri

**Affiliations:** Department of Urology and Organ Transplantation, University of Foggia, Foggia, Italy

**Keywords:** Medical research, Quality of life

## Abstract

The reason behind the spread of penis enlargement practices over time is rooted in the virility that the appearance of the genitals can give a man, as well as an altered perception of his own body. The approach should be to modulate the interventions on the real needs of patients, carefully evaluating the history, the psychological picture, and possible surgical advantages. The aim of this study was to shed light on cosmetic surgery of male genitalia through minimally invasive and more radical techniques, with the purpose of laying the foundation for possible indications and recommendations for the future. A non-systematic literature review using the PubMed and Scopus databases was conducted to retrieve papers written in English on cosmetic surgery of the penis published over the past 15 years. Papers discussing cosmetic surgery in patients with concomitant pathologies associated with sexual dysfunction were excluded. The main outcomes recorded were change in penile dimensions in term of length and girth and surgical complications.

## Introduction

The genital male organ is linked to an ancestral sense of man’s fertility and sexual performance, which are the reasons making it the main factor to affirm one’s own masculine identity and to procure pleasure in the partner [1]. Hence the sense of inadequacy and discomfort that many patients feel, even though from a physical-anatomical point of view they fall within the parameters of normality [2].

The reason behind the spread of penis enlargement practices over time is indeed rooted in the virility that the appearance of the genitals can give a man, as well as an altered perception of his own body. A large study of 25,594 healthy men found that 45% desired a larger penis [3]. Sometimes behind the request for penis enlargement, that is the increase of penile circumference, is hidden the desire to give more pleasure to the partner, although men generally view penis size as more important than women do [4]. In fact, the sexual pleasure of the woman can be physiologically generated by a sufficient distension of the mucosa of the vaginal canal, especially in the distal-third of the anterior vaginal wall were a significantly increased density of nerves and microvessels have been noted [5].

In this scenario, cosmetic surgery of the penis must be versatile complying with the various problems increasingly affecting this area of surgery. The key is to tailor interventions on the real needs of patients, carefully evaluating the history, the psychological background, and possible surgical advantages.

The following review aims to describe non-invasive and invasive approaches to cosmetic surgery of the penis with the purpose of laying the foundation for possible indications and recommendations for the future.

## Material and methods

A non-systematic literature review using the PubMed and Scopus databases was conducted to retrieve papers written in English on cosmetic surgery of the penis published over the past 15 years. The search was conducted in October 2021 using the following search strategy: “(((Penis) OR (penile)) OR (male genitalia)) AND ((Esthetic) OR (cosmetic)) AND (surgery)”.

Review articles, editorials, commentaries, and letters to the editor were included if deemed to contain relevant information on cosmetic surgery of the penis. References from selected articles were also assessed for inclusion. Papers discussing cosmetic surgery in patients with concomitant pathologies associated with sexual dysfunction were excluded.

The main outcomes recorded were changes in penile dimensions in term of length and girth and surgical complications. Flaccid, stretched and/or erect penile length as well as patients’ satisfaction rates were reported when available in the included studies. Results of individual studies were summarized and presented in tables as mean preoperative postoperative difference in penile length and girth. Two authors (A.A.C and U.G.F.) performed the initial screening of titles and abstracts independently to determine which papers could potentially meet the inclusion criteria. All authors finally agreed on the articles to include for discussion in the present review.

## Evidence synthesis

### Psychiatric background

Someone seeks male esthetic genital surgery for work, someone for pleasure and someone for real discomfort. Considering this, it is of pivotal importance to further investigate each patients true needs with an appropriate psychiatric and psychological assessment in order to find out the real impact of eventual treatments on their quality of life and to guide them with an appropriate pre-operatory counseling [6, 7].

Up to 66% and 12% of men in the general population perceive their penis size as average and smaller than average respectively and the desire to have a bigger penis was present in 46% of men who rated their penis as average and 91% of men who rated their penis as smaller than average [3]. The negative perception of the size of one’s own penis could underlie a somatoform disorder called *Penile Dysmorphic Disorder (PDD)* which is classified according to DMS-5, within *Body Dysmorphic Disorders (BDD)* [8]. Particularly, the PDD concerns the strong distress generated by a functional issue (patients dissatisfied with the erect size) or by the esthetic appearance (patients dissatisfied with the flaccid size) of the penis [9]. These patients often suffer from the “locker room syndrome,” which is a lack of self-confidence and fear of exposing their “small penis” in front of others, leading to social phobia [10]. The very same psychological reasons causing the patient to request surgery might also cause the patient to be dissatisfied even with the most successful surgical outcome [10].

Moreover, there is a group of men, with an anxiety disorder which does not comply with the BDD’s criteria, concerning excessive fear or worry of one’s genitalia being observed and negatively evaluated by others for the size: the Small Penis Anxiety (SPA) [11]. The degree of emotional distress and behavioral impairment linked to both these conditions can lead to the development of major depressive episodes, social anxiety or obsessive–compulsive disorder, thus causing a significant decrease in quality of life [9].

Interestingly, both PDD and SPA exclude men who present a true *micropenis*, described as a flaccid penis length <4 cm and an erect penis length <7.5 cm [12].

This suggests the psychiatric sphere must not be ignored before any treatment, because it sheds light to the reason leading most men to invasive surgery and on the right therapeutic option [13].

### Clinical evaluation

A complete clinical evaluation should always be performed prior to surgery, and it should include in addition to the psychiatric/psychosexual evaluation, a detailed medical history, an accurate physical examination with measurement of penis diameters [10], biochemical/sex hormone serum profiles and an ultrasound examination in flaccid and erect penis.

For lengthening surgery, the measurements of flaccid, stretched, and erect penis post pharmacological stimulation, are essential to have a quantitative idea of the possible gain for each patient. The stretched penile length represents the most overlapping measurement of the erect penis, corresponding to the distance between the pubic symphysis and the apex of the glans [14]. For enlargement surgery the circumference measurements of flaccid and erect penis, at the distal third of the shaft, just below the glans, at the middle third and at the proximal third at the level of the peno-pubic junction are important to evaluate a possible gain on girth.

Before planning any treatment, it is important to understand if the patient’s penis size is within the normal range, which corresponds for Caucasian man to a mean length of 9.16 (SD 1.57) cm for flaccid and 13.24 (SD 1.89) cm for stretched penis, an average circumference of 9.31 (SD 0.9) cm for flaccid and 11.66 (SD 1.1) cm for erect penis [15].

Clinical evaluation and the preliminary psychiatric evaluation can help in discerning those patients who would benefit from medical therapy or minimally invasive treatments from those who would benefit from surgery [7, 10].

**Fig. 1:**
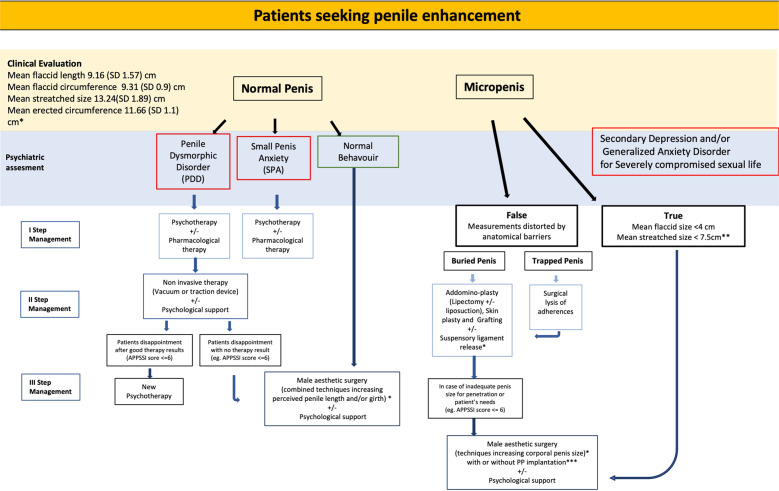
Psychiatric background and Management flowchart for patients seeking penile enhancement. *After complete counseling about complications and risk of failure. PDD: Penile Dysmorphic Disorder. SPA: Small Penis Anxiety. COPS-P: Cosmetic Procedure Screening Scale for PDD. CBT: Cognitive Behaviour Therapy. APPSSI: The Augmantation Phalloplasty Patient Selection and Satisfaction Inventory. QoL: Quality of Life. IIEF: International Index of Erectile Dysfunction.

An ideal flowchart for the diagnostic evaluation and management of patients requiring cosmetic genital surgery is presented in Fig. 1.

## Non-invasive treatment

### Vacuum device

The Vacuum device is a mechanical device that exploits a negative pressure created by a suction pump to draw blood from the circulation into the corpora cavernosa. It increases arterial flow, resulting in increased oxygenation of the corpora cavernosa and modulation of growth factors and apoptosis [16]. Although there is a large medical literature on vacuum devices for andro-rehabilitation of patients undergoing radical prostatectomy, there are few studies concerning its use for purely esthetic purposes [17].

Aghamir et al., in a prospective cohort study, programmed a 6-month therapy to 31 men, based on the use of the vacuum for 20 min, three times a week. The trial was unsuccessful, with only 11% of participants able to achieve at least 1 cm increase in stretched penile size on 8 month. The median stretched penile length increased from 7.6 (6.9–9.4) cm to 7.9 (7–9.7) cm but only 30% of patients were satisfied with the therapy and it was recorded a case of penile hematoma and a case of glans numbness [18].

Given the paucity of data, no evidence is available to confirm the real benefit of vacuum therapy for esthetic penile improvement (Table 1).

**Table 1:** Penile lengthening: non invasive treatment. A comparison between the outcomes from vacuum and traction therapy for penile lengthening, resulting from several studies.

	STUDIES	COHORT			LENGTHENING, mean cm increase (time)	GIRTHENING, mean cm increase (time)	COMPLICATIONS, Number
		*n*	Age, Mean (years)	Stretched Penile length, Mean cm			
VACUUM DEVICE	Aghamir et al. [18]	27	24	7.6	1 cm (8 months)	N/A	Glans numbness, 1 Hematoma penis, 1
	Abdel Raheem et al. 2010	31	51	13	0.5 cm (3 months)	N/A	Minor bruising, 2 Pump painful, 1
TRACTION DEVICE	Gontero et al. [20]	16	45.7	9.62	0.38 cm (6 months)	0.1 cm (6 months)	Pain and penile bruising, 1
	Nowroozi et al. [21]	44	30.1	11.2	1.3 cm (6 months)	0.2 cm (3 months)	Pain, 2 Bruising, 1 Glans numbness, 1
	Nikoobakht et al. [22]	23	26.5	11.56	1.7 cm (3 months)	0.2 cm (1 month)	N/A

### Traction therapy

A penile extender is a structure composed of two rings, one at the base of the penis and the other just below the glans, joined by metal rods, which are adjusted to hold the penis in traction, “stretching” it. The mechano-transduction signaling modulates gene expression, inhibiting apoptosis, stimulating cell proliferation, and modifying the extracellular matrix [19]. Over the years, it has been evaluated as a means of lengthening in place of or in combination to surgery to improve its results [20–22]. Gontero et al., in a prospective pilot study, evaluated the possibility of achieving a long-lasting increase in penile shaft length using the penile extender. Sixteen patients with PDD completed the 6-month therapy (daily use for at least 4 h), with a gain of 0.38 (0.02–0.73) cm on the stretched penis at sixth month and an insignificant gain on girth of 0.09 (0.24–0.05) cm. Moreover, they found progressive decline of the elongation obtained after the first month, probably linked to the shorter use of the device compared to that of the previous studies [20] Nowroozi et al., in a retrospective original article, administered the same traction therapy protocol to 54 patients with PDD, with 1.3 (±0.4) cm gained in stretched penis length at sixth month. 44 patients completed the treatment, with no evidence of decreasing in the periods off therapy. As far as the girth is concerned, it was reported a mean gain of 0.2 cm at 3 months. Moderate side effects have been reported in 4 patients: 2 with pain, 1 with glans numbness and another one with bruising [21]. Finally, Nikoobakht et al. reported outcomes of 23 patients complaining about short penis. Treatment with penile extender was continued for 3 months with progressive intensification from the first 2 weeks until the end of the third month. The mean stretched penile length was 11.5 (±1) cm at baseline and it increased to a mean of 12.4 (±1.39) cm and 13.2 (±1.47) cm respectively in the first and third month. No girth is gained in the proximal penis, while the glans circumference changed between the first and the second follow up, probably due to the device anchorage point. The authors did not report any side effect [22]. While there is concordance in the studies reporting the efficacy of these devices, prospective trials with larger patients’ cohorts and longer follow up is needed to evaluate the persistence of the results as well as the complications.

## Invasive treatments

### Penile lengthening surgery

Surgical treatments for penile lengthening can be divided into two main categories: *techniques that improve the perception of length* and *techniques that increase corporal length*, with or without penile prosthesis implantation.

### Techniques for improving perceived penile length

Usually, the management could be led by the presence or not of a specific clinical picture, like the acquired buried penis (ABP), which presents itself as a “false” micropenis [23, 24]. In fact, these patients should first undergo lipoplasty, and only in case of inadequate penis size a further intervention should be considered [25]. Until now a combination of multiple surgical approaches seems to be the most suitable solution for surgeons and patients.

### Lipoplasty

Lipoplasty is designed to improve the visibility of the shaft in obese patients and in patients with significant weight loss having excessive suprapubic fat and skin (“double belly” sign in orthostatic position) [23].

In all these patients it is very common an ABP, which could be defined as the entrapment of the penis by the surrounding tissue [25]. There is a slight difference between “buried” penis, defined as an hidden penis concealed by suprapubic fat, and the “trapped” penis, which presents a scarred or fibrous tissue, requiring surgical lysis and more invasive esthetic reconstruction procedures [26]. Surgical correction of an ABP can improve hygiene, urinary flow, sexual function and can reduce the risk of development of Squamous cell carcinoma of the penis, and for these reasons it represents the gold standard treatment [27]. The intervention develops in a series of steps, which should be evaluated case-by-case. A first approach could be a degloving incision to cut fibrous tissue on dartos or skin adhesions [23]. A *lipectomy* must then be performed, removing underlying adipose tissue and excess skin, being careful to ensure a tension-free closure, preventing the penis from telescoping back within the soprapubic tissue [28–30]. For a better cosmetic result several surgeons complete the lipectomy with the *liposuction*, which may precede or follow the first step [31]. The final step is represented by the closure of any skin defect with scrotoplasty [32] and, in case of large penile defects, with skin grafting vacuum assisted closure [25, 33].

In those patients with no ABP and a moderate pubic fat pad, surgeons could choose the liposuction to improve the perceived penile length [34]. The resection of the suspensory ligament may be associated in all patients undergoing a lipoplasty, after a pre-operatory counseling [35].

### Skin reconstruction plasty

Skin reconstruction plasty has been deployed to make the penile shaft more visible [36]. Today it represents the most commonly used method to access the suspensory apparatus and to fill the infrapubic space, although alternatives have been evaluated over the years [37].

*V-Y plasty:* this procedure involves the peno-pubic angle, but it was also used as an alternative for penoscrotal skin correction [38]. The first incision is an upside down “V”, which is subsequently closed as “Y”, lengthening the dorsal skin, and bringing the lateral skin medially [39]. Li et al. reports that the ideal inverted “V” should be at a 60° angle, because a greater angle may limit the amount of gainable length, while a shallower one may compromise vascularization of the flaps [40]. Major complications may be secondary to compromised flap vascularization during resection, thus wound dehiscence, infection, and/or dorsal flap loss [41]. Today it is not easy to understand how much visual gain is achieved from skin plasty only, as it is almost always associated with other techniques, firstly the suspensory ligament release [42].

*Z plasty*: this technique is another way of access to the suspensory ligament and can be used in case of cicatrix post circumcision with an high attachment of the skin on the penile shaft [43], as a prepuce sparing treatment in case of phimosis [44] and for scrotal reconstruction [45]. Scrotal raphe is taken as an incision site, on which the Z plasty is applied at a 60° angle allowing a 75% gain in visibility of the shaft. This technique is not easy to reproduce and exposes to the risk of circumferential choking of the penis and to high tension on the wound, causing poor blood supply and risk of breaking [45]. For these reasons the V-Y plasty is often preferred to this one [42].

*Flap reconstruction*: this method has been used mainly in patients with congenital micropenis secondary to epispadias. In these patients the dorsal skin may not be enough to cover the release of the corpora cavernosa after resection of the suspensory ligament [46]. Local rhomboidal skin flaps can be used with good esthetic results [46], or lateral scrotal flaps, whose rotation of the base allows bilateral coverage of the corresponding dorsal penile tract [47].

*Ventral Phalloplasty (VP):* this technique can be used to reduce visual penile shortening (buried penis appearance) secondary to high penile-scrotal skin insertion, generally after too aggressive circumcision or to improve perceived penile length in patients undergoing penile prosthesis implantation [48]. The scrotum is stretched away from the penis, two incisions are done, one parallel to the penis’ edge and the other convex to the scrotum’s edge, with the excision of excess scrotal skin. Miranda-sousa et al reported a penile length improvement after penile prosthesis implantation in 84% patients [49]. Similarly, Ahn et al., applied VP to improve perceived penile length as compensation for shortening after tunica albuginea plication. In this case, the penile skin is peeled away from Buck’s fascia until it reaches the penile–scrotal junction, where the Dartos and the scrotal septum are identified, the former being circumferentially peeled away from Buck’s fascia, the latter ventrally from the base of the penis. Eighty-seven percent of patients who underwent tunica albuginea plication combined with VP, reported an increase in perceived length after surgery [50].

*Scrotal reduction:* Few other techniques need to be mentioned for the treatment of adult patients consulting for bothersome scrotomegaly [51]. These patients may refer dissatisfaction of the appearance of their genitalia but usually present with discomfort while walking, using loose clothes, doing sports and during intercourse. Alter et al first described the surgical procedure. An horizontal excision of the mid to upper scrotum is performed to remove excessive scrotal skin. If there is unilateral scrotal enlargement, an asymmetrical excision of the lower scrotal skin may be performed. The dartos is then reapproximated with resorbable sutures, and the skin is closed with subcuticular resorbable sutures [34]. More recently, Lorenzo et al proposed a novel technique involving the excision of two rectangular skin flaps posteriorly along the perineum border of the scrotum up to the most dependent point of the scrotum. The Dartos fascia is then plicated and the skin is sutured posteriorly at the base of the scrotum [52]. Finally, a vertical skin resection along the median rafe with a subsequent Z plasty has been proposed in order to better preserve the scrotal sensitivity since the genital branch of the genitofemoral nerve and the ilioinguinal nerves run from lateral to medial [51].

### Suspensory ligament release

The release of the suspensory ligament, with or without the association of other procedures, has represented for many years the most commonly used penile lengthening technique [40]. The incision of the ligament allows the release of the corpora cavernosa from the pubic branches, changing the peno-pubic angle from acute to obtuse, giving the perception of a longer penis [53].

Borges et al recorded an improvement in penile length in 18 patients who underwent to penile prosthesis implantation combined with suspensory ligament incision: the mean flaccid penis gain was 2.43 (1.4–3.2)  cm, while the mean erect penis gain was 1.73 (1.1–2.2) cm [54]. Li et al confirmed this length gain, reporting an increase from 1 to 3 cm, but mostly with postoperative penile traction therapy [40]. Unfortunately, this technique exposes to several risks and complications, such as denervation and/or devascularization of the penis secondary to the resection of neurovascular bundles, lack of support and stability of the penile shaft with secondary difficulty in penetration during sexual intercourse, finally, a paradoxical complication is represented by the shortening of the penis due to the re-adhesion of the resected ligament flaps [41]. Indeed, to reduce the risk of this complication, several technique modifications have been proposed [55–57]. Bhavik et al., proposed to spare the suspensory ligament and resecting only the overlying fundiform ligament achieving similar results with lower risk of shaft shortening from ligament re-adhesion [56].

Another approach is based on filling the infra-pubic space resulting after ligament release between the pubic symphysis and the base of the penis [55]. Lipomatous tissue, testicular prosthesis, silicone, or dermal fat grafts may be used [57].

By using silicone and dermal fat grafts, Srinivas et al. demonstrated a 2.5 cm gain on penile shaft 6 months after ligament resection. However, few complications related to the use of synthetic products (herniation, foreign body reactions, infection, or erosion of surrounding tissues) have been described [57]. Another approach has been described by Zhang et al. using an acellular matrix of dermis in 15 adult men [55]. After liposuction of the pubic region, the procedure continues gaining access to the suspensory apparatus through sub-coronal circumcision, detachment of the penile skin from the deep fascia and subsequent complete resection of the subpubic ligament and partial resection of the suspensory ligament. The procedure is completed with the insertion of the dermal acellular matrix into the infra-pubic space. The result was an average gain of 2.4 (SD 0.8) cm at 3 months, with no reported complications and a high patients satisfaction rates [55].

### Techniques for increasing corporal penile length

More invasive surgical techniques up to total phalloplasty can be used to increase the effective length and width of the penis. These methods should be recommended in first instance to patients with true micropenis for whom the methods previously considered could be ineffective.

### Penile disassembly

In this original technique it was used a cartilage graft for length enhancement. After penile degloving, the neurovascular bundle is dissected dorsally and the corpus spongiosum ventrally, thus separating the corpora cavernosa from adjacent structures. A space is created in the most distal part of the shaft, separating the glans from the distal part of the corpora cavernosa, where an autologous rib cartilage graft is placed. Finally, the tissues are reassembled, covering the graft with the glans. Nineteen patients with micropenis underwent this procedure by Perovic and Djordjevic from 1995 to 1999, with an average gain in length of 2.5 cm in 13 patients and 3.5 cm in the other 6 and without evidence of iatrogenic injury to the urethra, cartilage extrusion, or erectile dysfunction at a median follow-up of 3.3 (1–4.5) years [58].

### Sliding elongation

*Sliding technique*: from 2012 to 2014, Rolle et al. designed and applied the sliding technique for increasing corporal penile length. Initially developed for patients with severe penile shortening secondary to Peyronie’s disease, this intervention is based on a double incision, one ventral and one dorsal. The length of these incisions is generally at least 4 cm and is based on the approximate stretching capacity of the neurovascular bundle and spongiosum. The tunica albuginea is then secured with absorbable suture and dorsal and ventral patch grafts are then placed over the residual defects. In patients who underwent such technique, the average length gain was between 2.5 and 4 cm [59]. Several technique modifications have been described [60] and nowadays sliding elongation is mainly used for increasing corporal penile length in patients with Peyronie’s disease. However, since it represents a challenging technique with associated surgical risks, its use should be avoided in patients with true micro penis and no underling pathologies in favor of less invasive techniques.

### Total phalloplasty

The effect of previously described techniques on penile length is, for most patients with micropenis, not enough to improve genital image and sexual quality of life.

With the achievements in the microsurgery field, free flaps incorporating sensory nerves have being used for the surgical treatment of micropenis. Total phalloplasty is a two-step procedure including the creation of a neophallus with an urethra that helps in urinating standing up and an acceptable esthetic size and sensation followed by penile prosthesis implantation to provide enough rigidity for sexual intercourse. Radial artery forearm free flap (RAFFF) phalloplasty was first described in 1984 and is still accepted as the standard technique for penile reconstruction worldwide [61]. Recently, Falcone et al. reported outcomes of 108 patients undergoing RAFFF. A primary anastomotic urethroplasty was performed in 90 patients (83.4%) and a staged procedure in the remainder. Four patients experienced an acute arterial thrombosis, leading to complete loss of the phallus in two. The most common complication was urethral stricture occurring in 49.1% of patients [62]. The anastomosis between the fixed and phallic part of the urethra is the most important stricture location and prone to fistulation in the early postoperative period. The authors also reported patient satisfaction with a five-point Likert scale ad hoc questionnaire to measure postoperative penile sensitivity. 80% of patients were satisfied postoperatively, 76% of patients managed to reach orgasm and reported an acceptable level of penile tactile and erogenous sensation. The results of this series concur with others in that the forearm free flap phalloplasty technique provides the patient with a satisfying surgical result, according to the patient and surgeon [63]. However, patients must be fully informed about possible limitations and complications of the technique, also on the long term, as to have realistic expectations [64].

### Techniques for increasing penile girth

The main goal of penile girthening surgery is to determine a long-lasting gain in the girth of both flaccid and erect penis improving the sexual quality of life.

Several methods have been adopted to achieve penile girthening, starting from the injection of the most various substances [65–67], the positioning of grafts and scaffolds [68, 69], up to more invasive methods such as cosmetic phalloplasty [70]. A summary of studies reporting outcomes of soft tissue filling, grafting and biodegradable scaffolds implantation for penile girthening, in presented in Table 2.

**Table 2:** Invasive treatment for penile enlargement. A comparison between the outcomes from soft tissue filling, grafting and biodegradable scaffolds implantation for penile girthening, resulting from several studies.

	STUDIES	*n*	Age (years)	Pre-operatory GIRTH	THERAPY	POST- OPERATORY GIRTH		SATISFACTION	COMPLICATIONS
SOFT TISSUE FILLERS	Kwak et al. 2010	41	42.5	At midshaft 7.48 (SD 0. 35) cm	20.56 ml of HA	+3.92, SD 0.25 cm (1 month)	+3.78, SD 0.26 cm (18 month)	18 months 100% patients satisfied (VAS)	Not reported
	Casavantes et al. 2015	203	37	Mid 10.54 (SD 1.49) cm	20 ml PMMA	Base 13 (SD 1.46) cm	Mid 12.76 (SD 1.41) cm	83%patients satisfied (grade 8–10)	-Nodules (52%) -Sensitivity decreased (2%) -Erectile function decreased (1.5%)
GRAFTS	Spyropoulos et al. [82]	5	30	Subcoronal 6.0 (SD 0.4) cm	Autologous Dermal fat graft	Base + 2.3, SD 0.25 cm	Subcoronal +2.6, SD 0.25 cm	7.54 (Post operatory APPSSI)	-Curvature with pain (25%) -Pain on erection and hypertrophic scar formation (75%)
	Alei et al. [86]	60	28.2	Flaccid 8.1 cm	Porcine dermal acellular matrix graft	Flaccid 11.3 cm	Erect 13.2 cm	98% patients satisfied (Post operatory APPSSI)	-Moderate fibrosis with minor retractions (6.21%) -Suture dehiscent (5.58%)
BIODEGRADABLE SCAFFOLDS	Djordjevic et al. [91]	21	28	Flaccid 11.6 (SD 0.8) cm	PLGA scaffold + Fibroblasts	Flaccid + 1.1, SD 0.4 cm	Erect +1.0, SD 0.3 cm	100% patients satisfied (Mark 3–5)	-Partial superficial necrosis (10%)
	Zhe Jin et al. 2010	69	33	Flaccid 8.18 (SD 0.83) cm Erect STUDIES	PLGA scaffold + Fibroblasts	Flaccid + 3.15, SD 0.42 cm	Erect +2.47, SD 0.49 cm	94.2% patients satisfied (VAS 3–10)	-Subcutaneous edema (2%) -Pinpoint erosion at the suture (2%)

*N* number of patients treated and evaluated on follow up, *Age* mean age of the cohort patients, *APPSSI* The Augmantation Phalloplasty Patient Selection and Satisfaction Inventory, *VAS* Visual Analogue Scale, *PMMA* polymethyl-methacrylate Microspheres, *HA* hyaluronic acid, *PLGA* poly-lactic-co-glycolic acid.

### Injection therapies

Soft tissue fillers are the second most widespread esthetic surgery technique in the United States of America for their reduced invasiveness, safety of use and low cost, counting almost 3 million procedures in 2018 [71].

### Silicone

Since its initial diffusion at the beginning of World War II, the safety of Silicon based injection has raised doubts in the competent authorities, to the point of reaching a suspension from the market in 1976 by the The U.S. Food and Drug Administration (FDA) [72]. These compounds determine granulomatous inflammatory reaction around silicone cyst-like vesicles with obliteration or dysfunction of the microcirculation [65]. Complications can be moderate, such as inflammation with severe edema or migration of injection fluid (liquid injection silicone, LIS), or very severe, such as penile shaft distortion with secondary erectile dysfunction, abscess formation, and even silicone pneumonitis, embolism, and/or multi-organ failure [66]. These reasons have generated, over the years, a vast medical literature on the surgical procedures available for the removal of silicone and the correction of resulting deformities [65–67].

### Fat

Autologous fat injection is based on the acquisition of fat by liposuction, its preparation and subcutaneous injection. A fair amount of literature on this procedure is available, witnessing complications and constant evolution over the years. Panfilov et al. injected up to 70 ml of body autologous fat through a preputial incision in 60 patients, 31 of which underwent to another injection at sixth month, with an average circumference gain of 2.65 (1.4–4) cm after 1 year [73]. Kang et al. reported similar outcomes in 52 patients, with a mean circumference gain of 2.5 cm, 6 months after surgery. The distal third thickness of the penis was 7.06 (SD 0.37) cm before treatment and 9.34 (SD 0.86) cm after treatment [74]. No noteworthy complication was reported in these two studies, and it is surprising how the circumference gained was preserved after several months, despite the exposure of adipocytes to resorption in a highly vascularized tissue. However, a new injection to preserve the thickness gained seems to be a common need. The most common complications are moderate, including pain, alteration of vibratory sensitivity, formation of residual fat nodules, skin deformities and scars [50], but a single non negligible case of death due to fat embolism after the injection of 70 ml of autologous fat has been reported in a 30-year-old man. [75].

The use of autologous fat is still an experimental method, patients should be informed on possible complications and about the need of new injections to preserve the thick gained.

### Soft tissue fillers

The use of soft tissue fillers for cosmetic purposes has steadily increased thanks to the mini-invasiveness and cost-effectiveness compared to more invasive procedures [2] and their versatility of use for various areas of the body [76]. The injected agents can be divided into resorbable (hyaluronic acid, HA) and non-resorbable fillers (polymethyl-methacrylate microparticles, PMMA) with different biochemical characteristics: the first (HA) has passive action while the second (PMMA) has delayed action but with bio-stimulating effect.

Soft tissue fillers are administered in four to six injections, divided equally on each side. Two lines parallel to the spongy body of the urethra are drawn as limiters of the area to be preserved. An automatic gun is used to inject precise volumes of filler, generally equal to 0.1 ml, between Buck’s fascia and the deepest part of the Dartos [77].

*Hyaluronic Acid (HA):* HA represents the most widely used long-lasting resorbable agent in cosmetic medicine. Its Biological characteristics make HA an ideal soft tissue filler because of non-migratory and long-lasting action due to its stability in the injection site, its relatively affordable cost, biocompatibility and non-antigenicity, thanks to which it does not cause inflammation or auto-immune reactions [78]. Thanks to its ability to bind water molecules it can maintain the volume acquired at the time of injection for additional months.

Kwak et al. subjected a penile injection of 20 ml of HA, obtaining a circumference improvement from the basal girth of 7.48 (SD 0.35) cm: 11.41 (SD 0.34) cm at 1 month and 11.26 (SD 0.33) cm until 18 months [79]. Sometimes patient satisfaction was affected by decreased sensitivity and stiffness of the shaft during erection, secondary to coverage of the corpora cavernosa by HA. No major complication was recorded in the study, but arterial embolization is mentioned in the medical literature concerning HA used in other tissues. This represent a possible complication, although it has not been reported in any case of penile injections [79].

*Polymethyl-methacrylate (PMMA) microspheres:* it is the most prominent exponent in the family of non-resorbable agents. Microparticles of polymethyl-methacrylate are suspended in solutions of bovine collagen or cellulose, and once injected lead to a granulomatous-like reaction in the injection tissue, which, consequently, is enriched with new supporting vascular tissue. They have been synthesized in recent years and have been already approved in several countries [80].

Casavantes et al. subjected 729 men to 2–3 sessions of injections, achieving not only an increase in average girth of 2.21 (SD 1.16) cm, but also an increase in average flaccid penis length of 0.7 cm, likely due to PMMA’s ability to create a stiffer shaft at rest. Eighty-three percent of patients were satisfied with the postoperative results [77]. Unfortunately, 52% of patients directly witnessed shaft deformities secondary to the formation of nodularities, single or multiple, and indentations due to areas of void. The development of granulomas has never been scientifically proven, but only confused with simple nodularities from inhomogeneous accumulation, since there is not enough immune reactivity between Dartos and Buck’s fascia for their formation [70].

With an attempt to shed light on possible differences between the two fillers, Yang et al. with a multi-center randomized study on 69 patients, compared the mean circumference outcomes at 6 and 18 months between the newly synthesized cross-linked HA and PMMA. The mean circumference gained by both the fillers didn’t differ more than 0.2 cm. However, the loss of this gain from 1 to 18 months was greater for the HA group than for the PMMA group (43% vs 21%) [80]. Moreover, excluding two cases of inflammation and three of pain in the site injection, no major complications were recorded. An interesting note is how satisfaction with sexual performance increases despite circumference gain is lost in the following months. There is often a psychological component in most patients requesting esthetic surgery and usually the strong psychological distress can be mitigated by these treatments [80].

Unfortunately, there are still few studies with long-term follow-up and with a complete evaluation of the sexual e psychological distress before and after treatment. Moreover, although several types of fillers have been introduced, only some are on the market in all countries [81].

### Grafting procedures

Penile grafting can be performed with autologous tissue or xenografts, generally from porcine or bovine [82, 83]. The former consists of dermis, which is important for vascular support and the survival of the graft itself, and subcutaneous fat that gives thickness to the treated tissue; while the latter consists of a acellular dermal matrix modified in the laboratory [82–84]. Today, acellular dermal matrix grafts are mainly used for this technique, characterized by excellent biocompatibility and chemical-physical characteristics that provide excellent rod stability, degradation and tensile strength [68]. The implantation can be realized in two ways through albugineal or peri-cavernosal surgery.

#### Albugineal grafting procedure

Albugineal grafting consists on the cavernous body thickening using saphenous-patch grafts or alloplastic materials [70]. This technique involves a peno-pubic incision up to Buck’s fascia, which is preserved, followed by complete degloving of the penis. One or two layers of dermis grafts or acellular dermal matrix are applied and sutured to the buck fascia, applying a compressive dressing at the end [85]. Austoni et al. was the first to use bilateral corporal venous grafts to expand corporal girth on 39 patients with normal penile diameter [70]. Bilateral longitudinal corporal incisions are made into the lateral aspect of the tunica albuginea from glans to pubis, where saphenous vein grafts are then placed for augmentation. This technique shows an average increase of erect penile diameter varying from 1.1 to 2.1 cm and no change in flaccid penis. Thus, should not be offered to men who are concerned with inadequate flaccid appearance, but rather to men who are motivated to augment erectile girth. Nowadays there are many techniques capable of earning girth, both in erect and flaccid penis, less invasive and of easier execution.

#### Pericavernosal grafting procedures

Pericavernosal grafting rely on subcutaneous enhancement around the corpora cavernosa.

Spyropoulos et al. used autologous graft transplantation on five patients with an average circumference increase of 2.3 (SD 0.25) cm in the proximal third of the penis and 2.6 (SD 0.25) cm in the distal third at a median follow up of 14 months (6–24) [82]. Alei et al. implanted xenograft porcine acellular dermal matrix on 69 patients with overlapping results in girth in both flaccid and erect penis at 1 year from surgery [86]

However, there is an important discrepancy between patient satisfaction declared in the questionnaires and the results obtained. Although major complications aren’t recorded, these procedures have given life to several problems. Edema, paraphimosis, seroma, and pain on erection are the most common, but are also reported deformities such as curvature and shortening of the shaft due to graft fibrosis, ulceration and necrosis of the skin, immune reactions against graft, infections and hypoesthesia [82, 86]. Xu et al. reported solutions to avoid these complications in a group of 78 patients who underwent penile girth enhancement with acellular dermal matrix [85]. The most frequent complication was preputial edema at 3–5 days after the procedure, avoidable with an infrapubic access to the shaft and a careful postoperative compression. Penile hematoma, exacerbated in hypertensive patients or by nocturnal erections, was rarer. The most serious complications were fibrosis, skin necrosis, and over-infection [85]. Necrosis may be secondary to delayed healing by traction mechanisms on the wound itself, and any circumferential skin loss could be resolved by using a scrotal flap reconstruction. Forty-seven patients reported discomfort at erection secondary to lack of mobility of the graft relative to the rest of the shaft, but only four of this reported dyspareunia, which forced surgeons to remove the patch. Twelve percent of patients were dissatisfied with the result obtained [85].

Given longer surgical times (from 3 to 5 h) even in expert hands, the high risk of moderate complications and patient dissatisfaction and the overall cost of the procedure, graft implantation for male esthetic surgery is not the wisest strategy to undertake.

### Biodegradable scaffolds

Although there are already several proofs about the use of biodegradable scaffolds for reconstructive surgery of urethra and corpora cavernosa [87, 88], in male esthetic surgery’s the evidence is still limited. The scaffolds are three-dimensional porous supports made of biocompatible and bioresorbable material able to promote cell adhesion and proliferation up to the formation of a new tissue and micro-network of blood circulation. Cells are first isolated from their natural biological environment, then cultured and implanted on scaffolds, made of a protein matrix capable of promoting their growth directly and indirectly [89–91]. Finally, the scaffolds are placed in the tissue where they degrade, leaving space for the extracellular matrix produced by the cells, thus restoring and implementing the tissue [69].

Perovic et al. used poly-lactic-co-glycolic acid (PLGA) scaffolds as a support for the proliferation of autologous fibroblasts, harvested from scrotal dermal tissue. They degloved the penis after subcoronal incision, and implanted scaffolds on the buck’s fascia near the urethra, without covering it. The average girth gained was of 3.15 cm and 2.47 cm in the flaccid and erect penis respectively, without serious complications and high satisfaction from patients [89]. Djordjevic et al. proved the feasibility of repeated treatment in 21 patients, who were unsatisfied with results achieved and wanted an additional girthening. The mean circumference gain was 1.1 (SD 0.4) cm in the flaccid and 1 (SD 0.3) cm in the erect penis after the second surgery at a mean follow-up of 38 months. There were two cases of necrosis of the overlying skin, which was treated conservatively, whereas no alterations in erection or sensitivity were noted with high satisfaction from patients [91]. Moreover, histological evaluation of what was seeded in the previous surgery revealed a high concentration of Ki-67 and vimentin as signs of cell proliferation, a new extracellular matrix formed by oriented collagen fibrils, a supporting vascular network and disparate fibroblasts and mast cells with probable regulatory activity [91]. Excellent results were drawn also from the study by Jin et al., who implanted high-interval porosity PLGA scaffolds in 69 patients, with an average circumference gain of 3.15 (SD 0.42) cm and 2.47 (SD 0.49) cm at 6 months, an overall preserved sexual function (IIEF-5 score > 22), a high degree of patient satisfaction and few complication [90].

The lack of comparative study with other procedures for penile enlargement and the necessity of skilled surgeons to achieve optimal results reported in the literature limit the widespread of this procedure.

### Subcutaneous penile implant

In the last years a new option for penile cosmetic surgery, a silicone penile implant called “Penuma^®^” has been approved and showed promising results. Penuma^®^ is a soft silicone subcutaneous implant placed, after infra-pubic access to the subcutis, on ¾ of the back of the penile shaft and secured to the glans with a polyester mesh [92]. The FDA issued a premarket notification for its use in the cosmetic correction of soft tissue deformities. Since then, the implant has been successfully used in thousands of men with optimal results. Elist et al reported outcomes of 400 patients who underwent Penuma implantation from 2009 to 2014. The results reported was a gain on midshaft circumference of 56.7%, from a mean preoperative girth of 8.5 ± 1.2 cm to a mean post-operative of 13.4 ± 1.9 cm, at a mean follow up of 4 years. There was also a gain on the penile flaccid length, from the pre-operative 9.1 ± 0.7 cm to the post-operative 11.3 ± 0.4 cm. Incidence of complications but with low incidence: 19 patients developed penile seroma (4.8%), requiring aspiration in seven patients (2%); 13 patients had wound infection (3.2%), of which eight required device removal (2%). 12 patients required implant removal: implant breakage with implant perforation and infection (1%), implant infection (1%), hematoma (0.25%), suture detachment (0.5%). Overall, Penuma^®^ achieved an high patients’ satisfaction rate. Using a non-validated but well-structured questionnaire (e.g., Augmentation Phalloplasty Patient Selection and Satisfaction Inventory “APPSSI”) the authors reported 81% of patients with high or very high levels of satisfaction [92].

From these results the Penuma® implants could achieve both girth and length enhancement with a high long-term satisfaction rate. Patient selection is the key to achieve optimal results and to ensure that all patients are fully informed and physically and mentally qualified, the authors recently published their patient selection protocol for the Penuma^®^ implant [93].

## Discussion

Patients seeking an improvement in the size of the penis can be divided into two main groups: patients with micropenis and patients with normal size penis. Among the first there are patients with a “true” micropenis and patients with a micropenis appearance, linked to other conditions like the *buried* or the *trapped* penis [26, 94]. Among the patients with normal size penis there are some with “Penile dysmorphic disorder” (PDD) or “small penis syndrome”, someone with “small penis anxiety” (SPA) and others without any problem related to their appearance but wishing to improve it [2, 9, 11, 70].

The best patient management may result from a complete clinical pre-operatory evaluation, including a psychiatric and psychosexual assessment, especially for patients who fall within the normal range diameters. For these patients the first step could be the differential diagnosis, through screening scales (eg. COPS-P), between patients with PDD and patients with SPA, to exclude the latter from an invasive management [9]. Moreover, in spite of the lack of evidence-based studies recommending psychotherapy for patients with penis size anxiety, there are studies showing how an appropriate counseling can dissuade these patients from surgical treatment [7] and how a cognitive behavior therapy could be beneficial for PDD patients [95, 96].

So, if surgery is still the gold standard treatment for patients with micropenis, today we do not have precise recommendations for patients with a normal penis size and PDD. It has long been known how cosmetic surgery may represent an important support to psychotherapy in mitigating or even resolving the psychological distress of patients not satisfied with their appearance [97, 98], although this way to heal psychiatric disorders could create a surgery addiction [99]. In the male esthetic surgery field, some studies report an increase in sexual satisfaction by patients who underwent enlargement procedures, whose real gain in shaft size was not preserved over time [80]. This testifies both the frequent presence of psychological distress and the real therapeutic possibilities of cosmetic genital surgery for these patients. In this scenario, the development and validation of questionnaires (e.g., Augmentation Phalloplasty Patient Selection and Satisfaction Inventory “APPSSI”) capable of evaluating patients’ motivations, based on sexual self-esteem and desire of penile augmentation, and expectations, may help surgeons in patient selection for augmentation penile surgery [6, 86].

Today, due to the constant media emphasis on esthetics and to the widespread diffusion of pornography and the continuous growth of the average age of survival and quality of life, cosmetic surgery of the genitals is a very lively field with an increasing demand on the world market [13, 100]. However, over time penile cosmetic surgery has not presented equal progress in all its fields, maybe because of an increasing attention to less invasive and cheaper methods, or because of a not always rigorous scientific method applied in studies. The second one is perhaps one of the major obstacles for a more rapid development, as the lack of objective or scientifically valid information does not allow to trace new paths or improve those already undertaken. On this matter it would be useful a structured follow-up based on objective measurements of outcomes and complications and based on the evaluation of patient satisfaction (eg. Sexual quality of life, Visual analogue scale, APSSI, Self-body esteem, etc.) through validated questionnaires.

A proper counseling must always be done before planning any intervention, to resize any false hopes built by the patient or indirectly induced.

Net of the patient’s psychiatric assessment, for those with a normal penis size, but seeking a permanent penile elongation, the wisest approach could be a combination of different methods, which improve the perceived penile length. The association between the V-Y skin advancement, the suspensory ligament release and the filling of the suprapubic space represents the best example of such synergy [37]. Filling the infrapubic space with acellular dermal matrix [55, 57] greatly reduces the risk of penile shortening due to the re-adhesion of the resected ligament flaps, while a partial sparing of the suspensory ligament could decrease the instability with secondary discomfort during sexual intercourse [41, 101]. In addition, lipoplasty could be functional in obese patients, with or without double-belly or a moderate pubic fat pad, giving a better cosmetic result with low risk of complications.

Regarding patients with micropenis, more invasive methods are advisable, with or without penile prosthesis implantation, depending on functional ultrasound evaluation. Among patients with a “false” micropenis (micropenis appearance), those with a *buried* penis could undergo a structured management, including lipectomy, liposuction and skin plasty, with or without suspensory ligament release and grafting of the skin loss, while those with *trapped* penis could undergo the same management, previous surgical lysis of the adherence and an accurate phalloplasty [26–28, 31–33]. For patients with true micropenis the sliding technique could be a good solution but the RAFFF phalloplasty is accepted as the standard technique for best results in penile reconstruction [62].

Among non-invasive treatments, we analyzed several studies on vacuum and traction therapy. They can make a gain in length, but with moderate and time-limited results, although without major complications [18, 20–22, 102]. It would be interesting to know if this non-invasive management can meet the needs of patients with a normal-sized penis, mitigating psychiatric disorders in those who suffer from it.

Regarding penile enlargement, there are no guidelines, nor a standardization of the procedures so far recounted in the literature. Over the years, several filling substances have been used including paraffin, vaseline, mineral oil, cod liver oil, metallic mercury, and petroleum jelly. These substances could cause a foreign body reaction leading to penile scarring and deformity, abscess formation, ulceration, and erectile dysfunction [103]. Although all such problems are well known, self-injections remain a common means of increasing penile girth for Eastern Europe and Eastern Asia people [104]. Reasons for this unreasonable practice include, on one hand, the fact that it is easily available and easily performed by non-medical personnel or the patient himself, on the other hand, lack of standardized medical or surgical techniques for penile girth enhancement. More and less invasive methods have been developed over time [72, 73]. Today, the use of soft tissue fillers has rapidly spread, thanks to the inexpensiveness and simplicity of the method compared to other procedures [76]. The results obtained with hyaluronic acid [79] and PMMA [70] in terms of circumference gained are very interesting, avoiding most of the historic complications of this surgery. However, the procedure, although not excessively invasive, requires expert surgeons, as well as further booster treatments for those who wish to maintain over time the circumference gained, making it much more expensive than it could be. Moreover, even if several types of fillers have been introduced, only some are on the market in all countries [81] and longer follow up studies are still necessary to draw definitive conclusions and include them in future guidelines.

Interesting prospects come from the biodegradable scaffolds for the generation of new layers of tissue. Despite the excellent results demonstrated through histological analysis of the tissues formed [91], tissue engineering is expensive, complex, and invasive compared to soft tissue fillers. Additionally, evidence is still limited to few case reports, and we are far from being able to draw conclusions.

The present review has some limitations due to inherent bias of the existing literature. First, experiences of skilled surgeons operating on a large cohort of patients are reported, which makes it difficult for us to understand whether the various techniques can be reproduced by less experienced hands. Secondly, statistical data about the outcomes of all surgical procedures are not always available, and it is not unusual to note inconsistencies between the complications arising after surgery and patient’s satisfaction data reported by the authors. Third, randomized control studies and comparative studies are lacking thus preventing us from giving any clear-cut recommendations however the aim of the present study was to suggests gaps in the available evidence and strategies to obtain those answers that are currently missing and that are necessary to develop guidelines for cosmetic surgery of the male genitals.

## Conclusions

The review of the literature on penile esthetic surgery, shed lights on the unmet needs for patients who request it. The available evidence is limited to single surgeon case series and randomized trials are lacking. A psycho-physical evaluation along with accurate patients’ selection and counseling are mandatory steps to achieve optimal outcomes. A multidisciplinary approach is often necessary, especially with patients with psychiatric disorders, to better assess patients’ eligibility for these kinds of treatments and eventually fulfill patients’ expectations.

## Data Availability

The authors confirm that the data supporting the findings of this study are available within the article.
